# Baicalein Alleviates Iron Overload-Induced Ferroptosis and Osteogenic Blockade in Osteoblasts by Activating the Nrf2/GPX4 Pathway

**DOI:** 10.34133/bmef.0230

**Published:** 2026-02-05

**Authors:** Zengfeng Guo, Ningfeng Zhang, Junshen Huang, Wang Zhang, Yawei Hu, Shaochu Chen, Ming Gong, Jianhua Zhou, Jiancheng Yang, Jiawen Wu

**Affiliations:** ^1^ Guangzhou University of Chinese Medicine, Guangzhou 510006, Guangdong, China.; ^2^ Department of Spine Surgery, People’s Hospital of Longhua, Shenzhen 518109, Guangdong, China.; ^3^ Department of Osteoporosis, Honghui Hospital, Xi’an Jiaotong University, Xi’an 710054, Shaanxi, China.

## Abstract

**Objective:** This study aimed to investigate the protective effects and underlying mechanisms of baicalein against iron overload-induced osteoblast dysfunction and bone loss. **Impact Statement:** This research is the first to demonstrate that baicalein, a natural flavonoid, functions as a dual-action agent combining iron chelation and antioxidation to prevent iron overload-induced ferroptosis in osteoblasts, offering a novel therapeutic strategy for iron overload-related osteoporosis. **Introduction:** Iron overload contributes to osteoblast damage and osteoporosis through ferroptosis, an iron-dependent cell death pathway. Current treatments fail to simultaneously address iron accumulation and bone loss, highlighting the need for effective dual-function therapies. **Methods:** Using iron dextran-treated MC3T3-E1 osteoblasts and a murine iron overload model, we assessed the effects of baicalein on cell viability, osteogenic differentiation, ferroptosis markers, and the nuclear factor erythroid 2-related factor 2 (Nrf2)/glutathione peroxidase 4 (GPX4) pathway via biochemical assays, Western blot, and micro-computed tomography. Genetic and pharmacological inhibition of Nrf2 were applied to validate the mechanism. **Results:** Baicalein chelated iron, scavenged reactive oxygen species, and suppressed ferroptosis in osteoblasts, restoring differentiation under iron overload. It activated Nrf2 nuclear translocation and upregulated GPX4/solute carrier family 7-member 11 (SLC7A11) expression. In mice, baicalein reduced iron deposition, oxidative stress, and bone loss, and these effects were abolished by Nrf2 inhibition. **Conclusion:** Baicalein alleviates iron overload-induced osteoblast ferroptosis and osteoporosis by activating the Nrf2/GPX4 pathway, supporting its clinical potential as a therapeutic agent for iron-related bone disorders.

## Introduction

Iron overload conditions, also known as hemochromatosis, represent a variety of diseases that lead to systemic iron overload and organ damage, which can be classified as primary and secondary [1]. Hereditary hemochromatosis, an inherited iron overload disorder due to the deficiency of hepcidin, is the most common primary hemochromatosis [2]. Secondary iron overload is common in hemoglobinopathies, including thalassemia [3], sickle cell disease [4], and myelodysplastic syndromes [5], and is indirectly induced by ineffective erythropoiesis and regular blood transfusion therapy. An increasing number of case reports, clinical studies, and animal models revealed that osteoporosis/osteopenia is one of the common features of iron overload disorders.

Currently, the primary treatments for iron overload-related bone disorders include iron chelators, phlebotomy, and anti-resorptive medications [6–8]. However, these strategies are associated with substantial limitations. For instance, deferoxamine requires prolonged infusion, leading to poor patient compliance; deferiprone carries risks of agranulocytosis and neutropenia; and deferasirox may cause renal/hepatic toxicity and adverse gastrointestinal effects [9]. Phlebotomy itself is an invasive procedure and can exacerbate the condition in anemic patients. Although anti-resorptive drugs such as bisphosphonates are effective in treating osteoporosis [10], they only provide symptomatic relief without addressing the underlying cause. Therefore, there is an urgent need to develop dual-function agents that can both chelate iron and treat osteoporosis effectively.

Baicalein, a natural flavonoid derived from the traditional Chinese medicine *Scutellaria baicalensis*, is one of the key bioactive compounds responsible for its pharmacological effects. Studies have confirmed that baicalein promotes osteoblast differentiation and bone formation while inhibiting osteoclastogenesis and bone resorption, thereby maintaining bone homeostasis and exerting anti-osteoporotic effects [11–13]. Importantly, baicalein serves as an excellent iron chelator. As early as 1996, Gao et al. [14] demonstrated in vitro that baicalein forms complexes with iron, thereby inhibiting lipid peroxidation. Pérez et al. [15] systematically evaluated the iron-chelating properties of baicalein in vitro and found that a 2:1 baicalein–iron complex readily forms in 20 mM phosphate buffer (pH 7.2). Further analysis revealed that the iron-binding site of baicalein is located at the O6/O7 oxygen atoms on the A-ring. In vivo, several studies have demonstrated that baicalein effectively reduces iron accumulation in mouse models of iron overload [16–19]. Additionally, baicalein acts as a potent free radical scavenger, further mitigating iron overload-induced oxidative stress damage [20–22]. Thus, baicalein represents a promising dual-function agent capable of both chelating iron and treating osteoporosis.

Ferroptosis, an iron-dependent form of regulated cell death, is considered a major mechanism through which iron overload induces cell death [23,24]. Studies have revealed that iron overload can trigger ferroptosis in osteoblasts, resulting in reduced bone formation and accelerated bone loss [25–27]. Conversely, inhibition of osteoblastic ferroptosis has been shown to mitigate impaired osteogenesis and prevent the development of osteoporosis [28,29]. In particular, recent reviews suggest that targeting ferroptosis may represent a promising therapeutic strategy for age-related bone disorders [30]. Although several studies have indicated that baicalein possesses antiferroptotic potential, its ability to suppress iron overload-induced ferroptosis in osteoblasts remains unclear. Therefore, in this study, using models of iron overload-induced ferroptosis and osteoporosis, we comprehensively investigated the protective effects and underlying mechanisms of baicalein against osteoblastic ferroptosis and bone deterioration. This work highlights the potential of baicalein as a promising ferroptosis inhibitor and its considerable therapeutic value in the treatment of iron overload-associated bone diseases.

## Results

### Baicalein inhibited the iron overload-induced decrease in osteoblast viability and differentiation

**Fig. 1.**
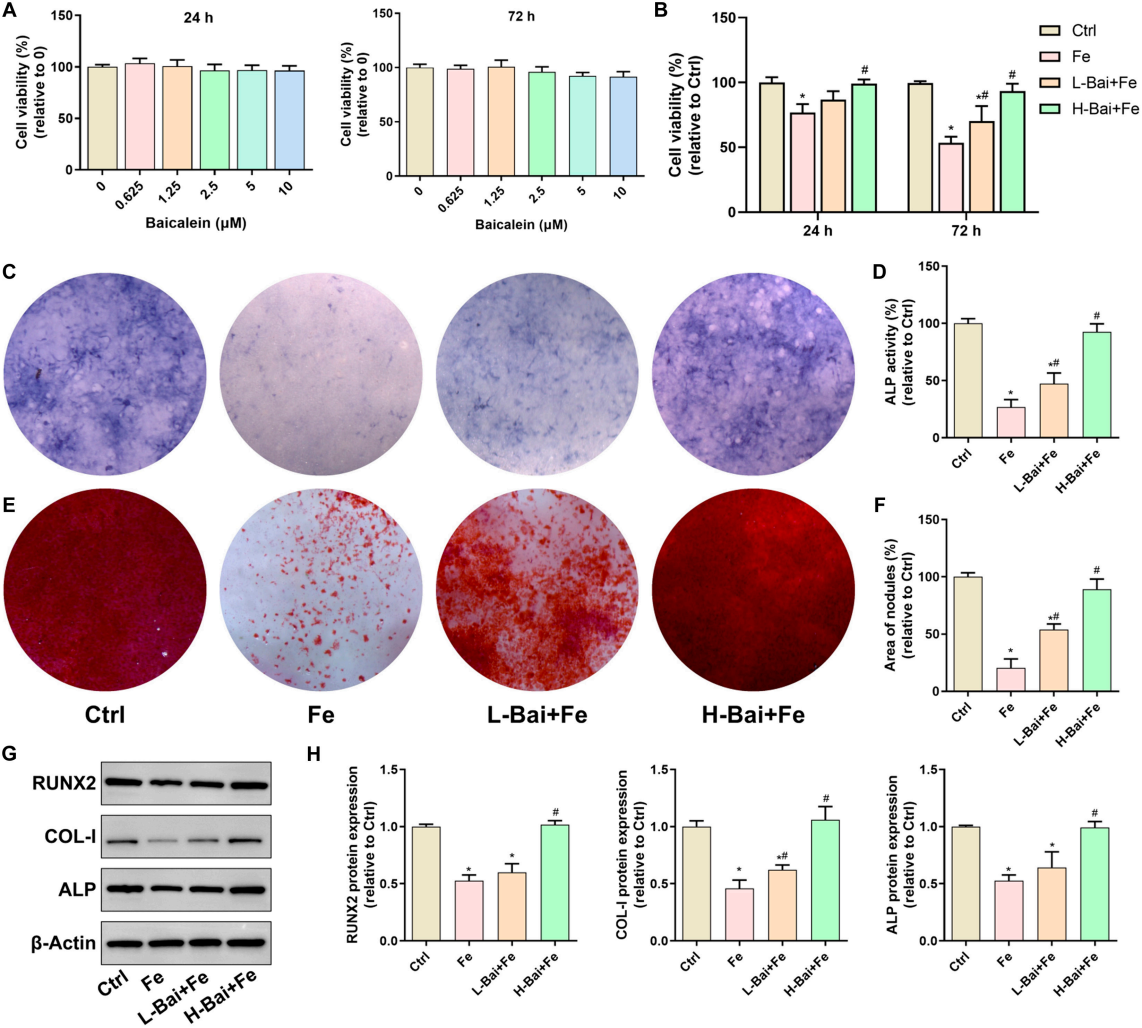
Evaluation of the effects of baicalein on the proliferation and differentiation of osteoblasts under excess iron exposure. (A) Cell viability of osteoblasts treated with different concentrations of baicalein for 24 and 72 h. (B) Effect of a low concentration of baicalein (L-Bai, 1 μM) and a high of concentration baicalein (H-Bai, 10 μM) on osteoblast viability under iron overload. (C) Alkaline phosphatase (ALP) staining after 7 d of osteogenic differentiation induction. (D) ALP activity after 7 d of osteogenic differentiation induction. (E) Alizarin Red S staining after 21 d of osteogenic differentiation induction. (F) Quantitative analysis of the mineralized nodule area. (G) Protein expression related to osteogenic differentiation as detected by Western blot, including runt-related transcription factor 2 (RUNX2), collagen I (COL-I), and ALP. (H) Quantitative analysis of protein band gray values. *P < 0.05 vs. control group (Ctrl); ^#^*P* < 0.05 vs. Fe.

Cell Counting Kit-8 assays revealed that baicalein at concentrations ≤10 μM had no effect on osteoblast viability (Fig. 1A). Iron dextran (50 μM) significantly decreased osteoblast viability, and this effect was reversed by additional supplementation with baicalein (Fig. 1B). Excess iron markedly inhibited the osteogenic differentiation of osteoblasts, as evidenced by decreased alkaline phosphatase (ALP) activity (Fig. 1C and D), reduced formation of mineralized nodules (Fig. 1E and F), and down-regulated expression of differentiation-related proteins, including collagen I (COL-I), ALP, and runt-related transcription factor 2 (RUNX2) (Fig. 1G and H). Baicalein, particularly at higher concentrations (10 μM), significantly alleviated iron-suppressed osteogenesis.

### Baicalein reduced the intracellular Fe^2+^ content and ROS levels

**Fig. 2.**
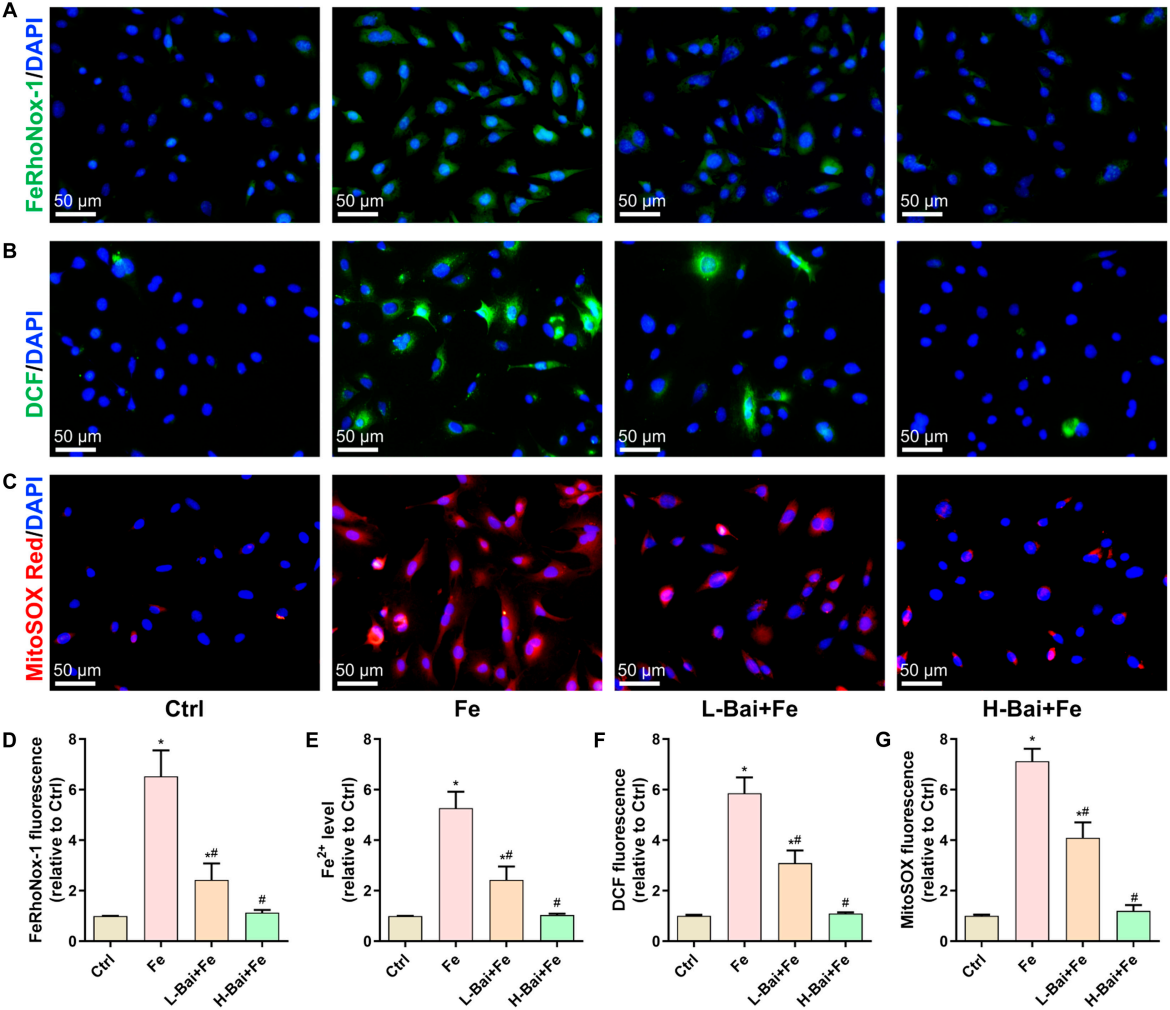
Evaluation of the effects of a low concentration of baicalein (L-Bai) and a high concentration of baicalein (H-Bai) on reactive oxygen species (ROS) and iron content in osteoblasts under excess iron exposure. (A) Changes in intracellular ferrous iron (Fe^2+^) levels as observed by FeRhoNox-1 fluorescent probe staining. (B) Changes in total cellular ROS levels as detected by 2′,7′-dichlorodihydrofluorescein diacetate (DCFH-DA) fluorescent probe staining. (C) Changes in mitochondrial ROS levels as measured by MitoSOX Red fluorescent probe staining. (D) Quantitative analysis of the fluorescence intensity of Fe^2+^. (E) Detection of intracellular Fe^2+^ concentration. (F) Quantitative analysis of total cellular ROS fluorescence intensity. (G) Quantitative analysis of mitochondrial ROS fluorescence intensity. *P < 0.05 vs. Ctrl; ^#^*P* < 0.05 vs. Fe. DAPI, 4′,6-diamidino-2-phenylindole; DCF, dichlorodihydrofluorescein.

The study found that baicalein possesses excellent iron-chelating and reactive oxygen species (ROS)-scavenging capabilities. Staining with the FeRhoNox-1 probe and measurement of intracellular Fe^2+^ content showed that excess iron stimulation increased intracellular Fe^2+^ levels, which were significantly reduced by baicalein treatment (Fig. 2A, D, and E). 2′,7′-Dichlorodihydrofluorescein diacetate (DCFH-DA) staining showed that iron overload induced a significant increase in intracellular dichlorodihydrofluorescein signal, and baicalein reduced intracellular ROS levels in a concentration-dependent manner (Fig. 2B and F). Consistently, baicalein also significantly mitigated the increase in mitochondrial superoxide (labeled by MitoSOX Red) in iron dextran-treated cells (Fig. 2C and G).

### Baicalein inhibited iron overload-induced ferroptosis

**Fig. 3.**
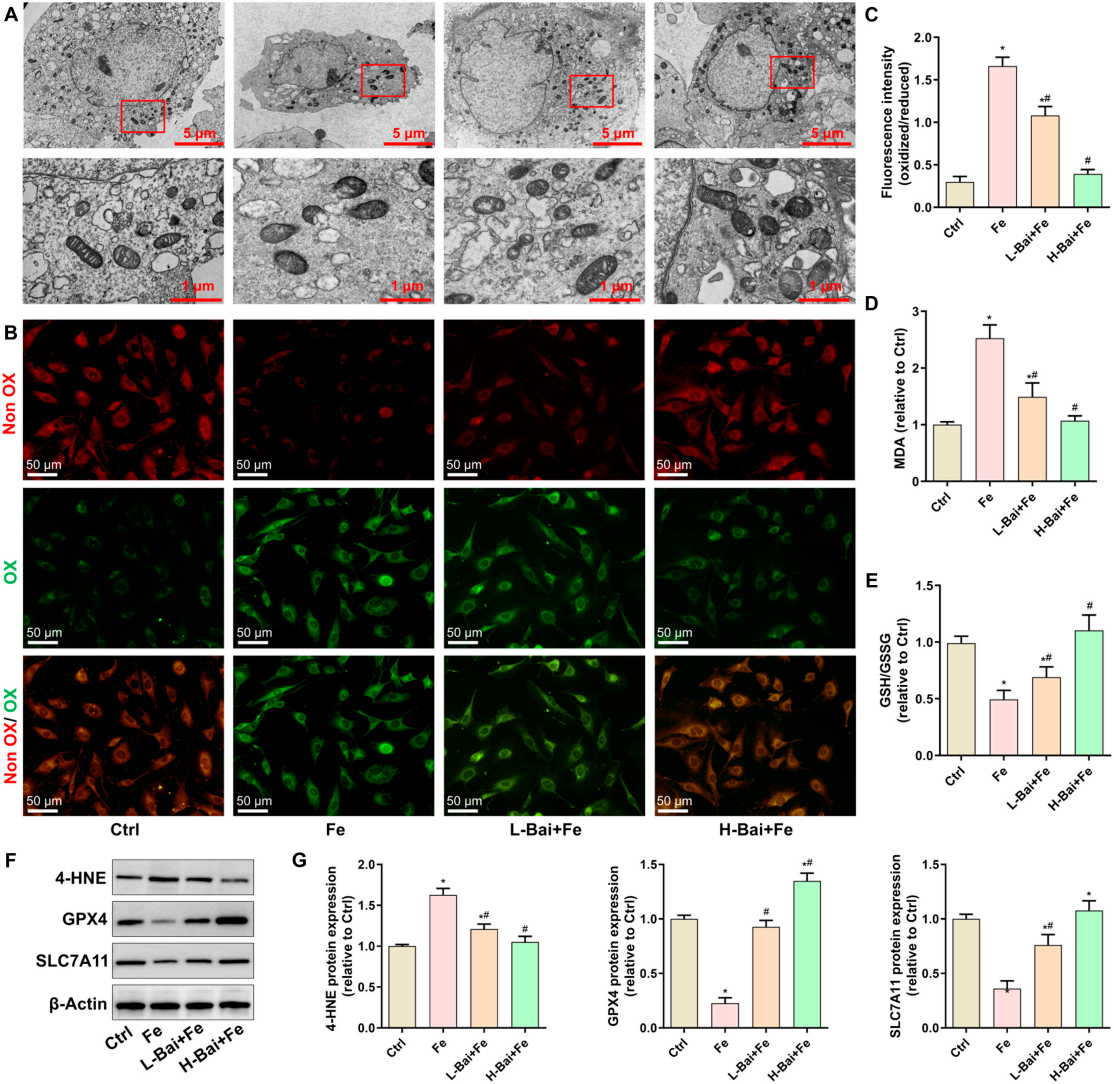
Evaluation of the effects of a low concentration of baicalein (L-Bai) and a high concentration of baicalein (H-Bai) on ferroptosis in osteoblasts under excess iron exposure. (A) Mitochondrial morphology was observed by transmission electron microscopy (TEM). (B) Lipid peroxidation levels were assessed using C11 BODIPY fluorescent probe staining. (C) Quantitative analysis of the ratio of oxidized to reduced lipids. (D) Detection of intracellular malondialdehyde (MDA) levels. (E) Assessment of the ratio of reduced glutathione (GSH) to oxidized glutathione (GSSG). (F) The expression of ferroptosis-related proteins was detected by Western blot. (G) Quantitative analysis of protein band gray values. *P < 0.05 vs. Ctrl; ^#^*P* < 0.05 vs. Fe. OX, oxidized; Non OX, nonoxidized; 4-HNE, 4-hydroxynonenal; GPX4, glutathione peroxidase 4; SLC7A11, solute carrier family 7-member 11.

Given that iron is a key trigger of ferroptosis, we evaluated the effect of baicalein on osteoblast ferroptosis. Transmission electron microscopy images of osteoblasts revealed that iron dextran induced mitochondrial crest disappearance and membrane rupture (Fig. 3A). Baicalein treatment ameliorated the iron dextran-induced changes in mitochondrial morphology. Detection of lipid peroxidation using C11 BODIPY demonstrated that iron dextran increased lipid peroxidation, while baicalein decreased the fluorescence intensity of oxidized (OX) C11 BODIPY (Fig. 3B and C). Consistently, baicalein suppressed the iron-induced increase in cellular malondialdehyde (MDA) levels (Fig. 3D). Conversely, the iron overload-induced decrease in the reduced glutathione (GSH)/oxidized glutathione (GSSG) ratio was reversed by baicalein (Fig. 3E). Western blot analysis revealed that baicalein supplementation concentration-dependently inhibited the iron-induced upregulation of 4-hydroxynonenal (4-HNE) and down-regulation of glutathione peroxidase 4 (GPX4) and solute carrier family 7-member 11 (SLC7A11) (Fig. 3F and G). These results indicate that baicalein acted as a ferroptosis inhibitor in cells exposed to excess iron.

### Baicalein inhibited iron overload-induced ferroptosis and impaired osteogenesis by activating the Nrf2 pathway

**Fig. 4.**
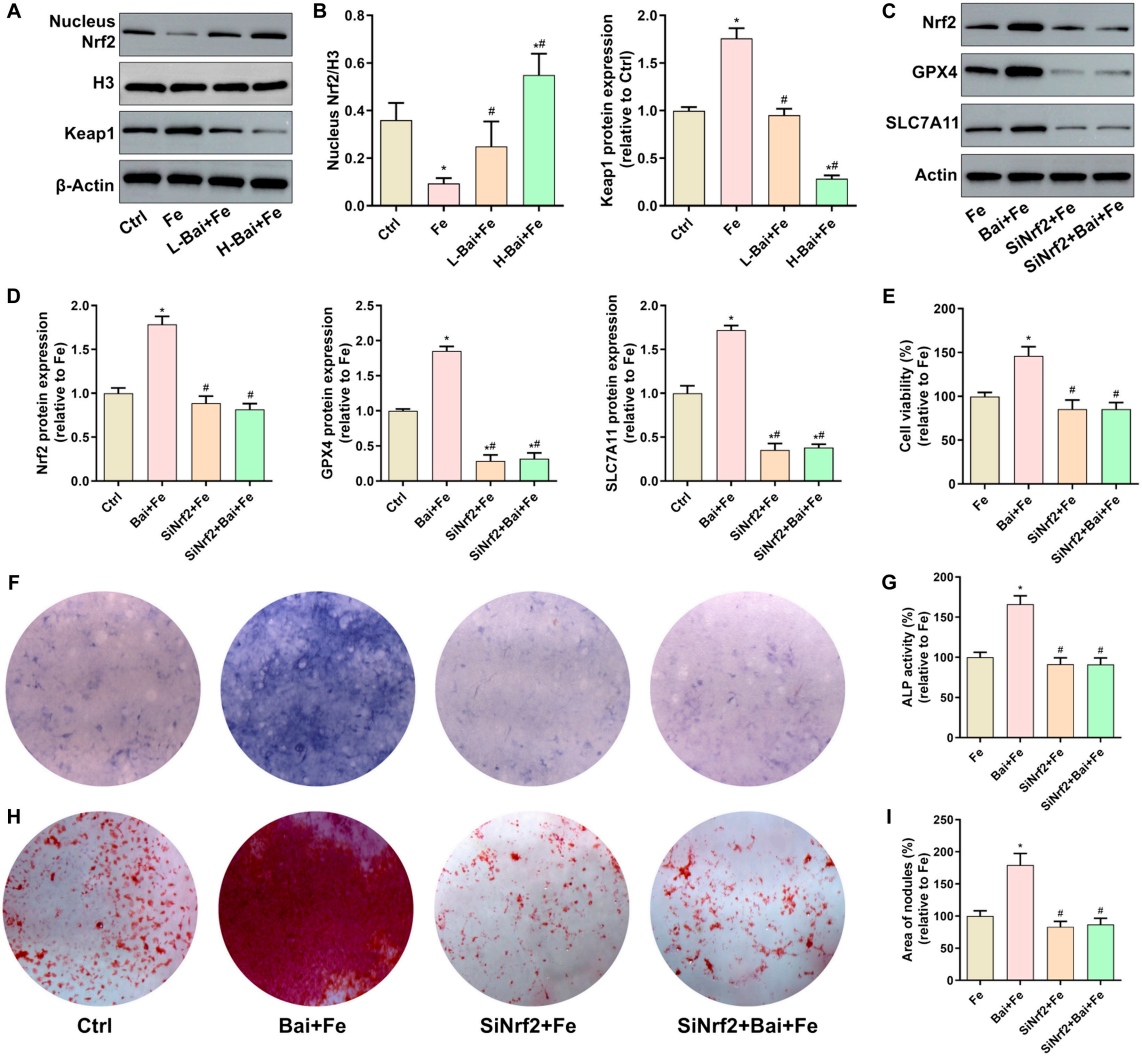
Evaluation of the effects of baicalein on the nuclear factor erythroid 2-related factor 2 (Nrf2)/GPX4 pathway in osteoblasts under excess iron exposure. (A) Protein expression of nuclear Nrf2 and total Kelch-like ECH-associated protein 1 (Keap1) detected by Western blot. (B) Quantitative analysis of the gray values of nuclear Nrf2 and Keap1 protein bands. (C) Protein expression of Nrf2, GPX4, and SLC7A11. (D) Quantitative analysis of the gray values of Nrf2, GPX4, and SLC7A11 protein bands. (E) Evaluation of osteoblast viability after 48 h of treatment. (F) ALP staining after 7 d of osteogenic differentiation induction. (G) Assessment of ALP activity after 7 d of osteogenic differentiation. (H) Alizarin Red S staining after 21 d of osteogenic differentiation induction. (I) Quantitative analysis of the mineralized nodule area. *P < 0.05 vs. Ctrl; ^#^*P* < 0.05 vs. Fe in (A) and (B); ^#^*P* < 0.05 vs. Bai+Fe in (C) to (I). siNrf2, small interfering RNA targeting Nrf2.

Mechanistically, given the crucial role of the Kelch-like ECH-associated protein 1 (Keap1)/nuclear factor erythroid 2-related factor 2 (Nrf2) signaling pathway in lipid peroxidation and ferroptosis, we investigated the regulation of this pathway by baicalein. The results showed that excess iron inhibited nuclear translocation of Nrf2 and promoted Keap1 expression, which was restored by baicalein intervention (Fig. 4A and B). To further confirm the role of Nrf2 signaling in the cytoprotective effects of baicalein, we constructed Nrf2-knockdown small interfering RNA (siRNA). We found that the knockdown of Nrf2 abolished the ability of baicalein to reverse the iron dextran-induced upregulation of 4-HNE expression and down-regulation of GPX4 and SLC7A11 expression (Fig. 4C and D). Phenotypically, Nrf2 knockdown prevented baicalein from inhibiting the iron overload-induced decreases in cell viability (Fig. 4E), ALP activity (Fig. 4G), and mineralized nodule formation (Fig. 4H and I). These results suggest that baicalein promotes osteogenic differentiation by inhibiting iron overload-induced ferroptosis via activation of the Nrf2/GPX4 pathway.

### Baicalein prevented iron accumulation-induced bone loss and inhibition of bone formation

**Fig. 5.**
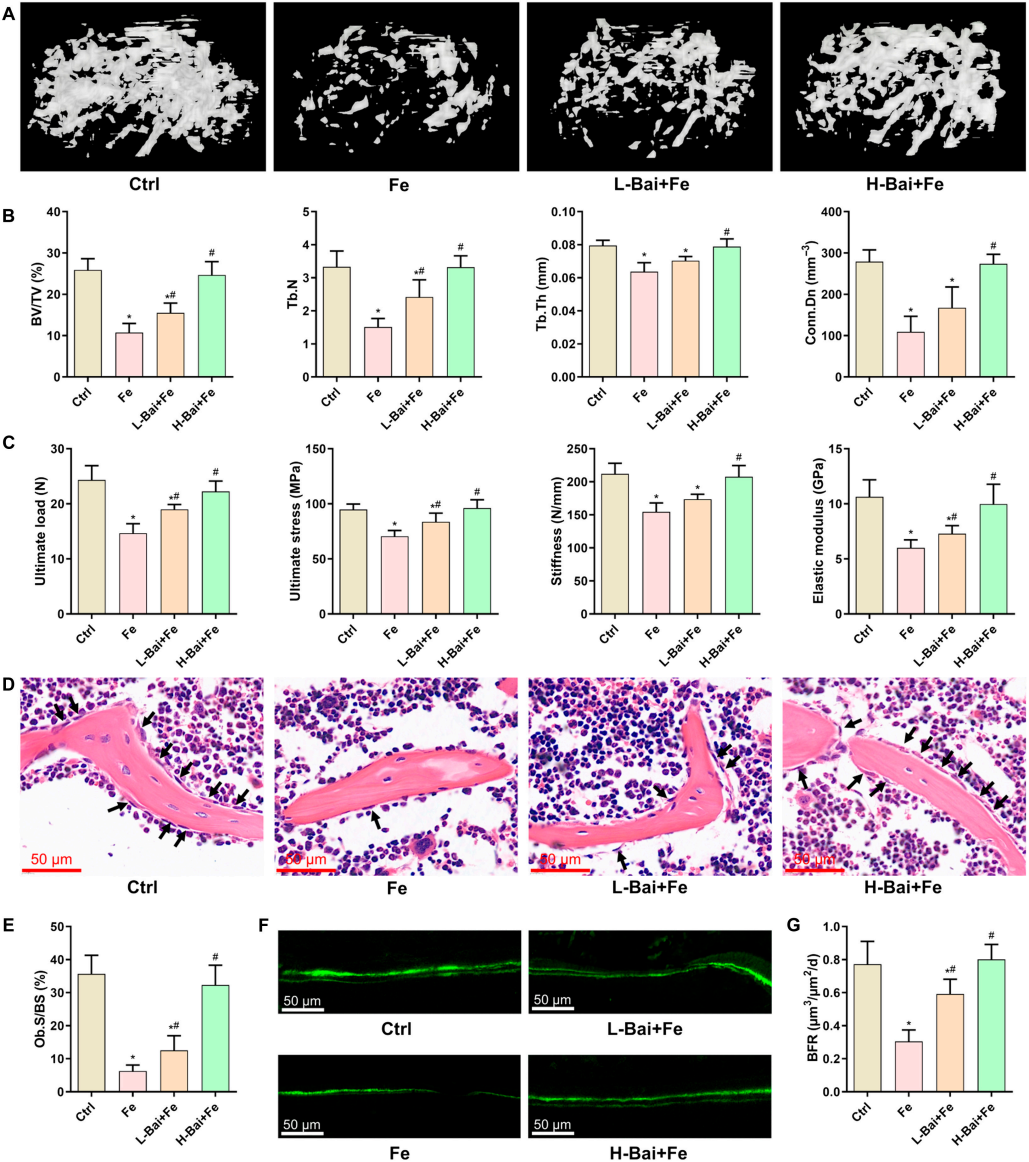
Evaluation of the effects of a low dose of baicalein (L-Bai) and a high dose of baicalein (H-Bai) on iron overload-induced bone loss in mice. (A) Three-dimensional reconstruction images of the trabecular bone in the mouse femur. (B) Quantitative analysis of the microarchitectural parameters of femoral trabecular bone. (C) Analysis of the mechanical strength of the mouse tibia. (D) Observation of osteoblasts on the bone surface by hematoxylin and eosin (H&E) staining; osteoblasts are marked by black arrows. (E) Quantitative analysis of osteoblast surface per bone surface (Ob.S/BS). (F) Evaluation of bone formation using calcein double labeling. (G) Quantitative analysis of the bone formation rate (BFR). *P < 0.05 vs. Ctrl; ^#^*P* < 0.05 vs. Fe. BV/TV, bone volume fraction; Tb.Th, trabecular thickness; Tb.N, trabecular number; Conn.Dn, connectivity density.

In vivo, we used gavage administration to elucidate the effect of baicalein on iron overload-induced bone loss. Representative micro-computed tomography (micro-CT) images showed significant destruction of the bone structure in the femurs from iron-overloaded mice (Fig. 5A). However, the bone structure was markedly improved in the baicalein treatment groups, especially the high-dose group. Quantitative analysis of bone structural parameters indicated that compared with the control group, the model group exhibited significant decreases in bone volume fraction (BV/TV), trabecular thickness (Tb.Th), trabecular number (Tb.N), and connectivity density (Conn.Dn). These changes in structural parameters were completely reversed by a high dose of baicalein (Fig. 5B). Three-point bending tests showed that iron overload led to reductions in the ultimate load, ultimate stress, stiffness, and elastic modulus of the tibia, and baicalein significantly inhibited the attenuation of bone strength in iron-overloaded mice (Fig. 5C). These results indicate that baicalein ameliorated iron overload-induced bone loss and reduced bone strength. Hematoxylin and eosin staining results showed that the injection of iron dextran led to a significant decrease in the number of osteoblasts on the bone surface, which was increased by baicalein treatment in iron-overloaded mice (Fig. 5D and E). Calcein labeling revealed that excess iron significantly inhibited the bone formation rate per bone surface (BFR/BS), and this was restored by a high dose of baicalein (Fig. 5F and G).

### Baicalein alleviated iron overload-induced iron accumulation and oxidative stress in mice

**Fig. 6.**
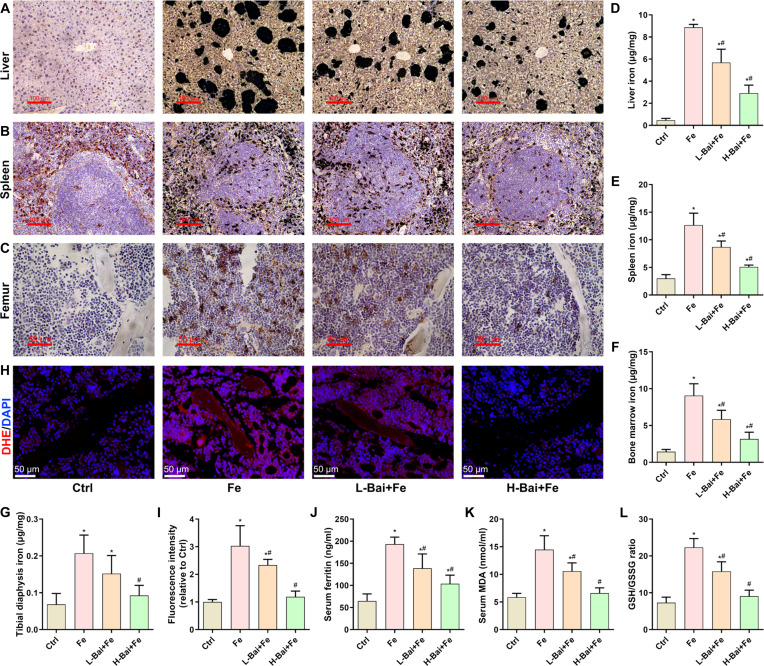
Evaluation of the effects of a low dose of baicalein (L-Bai) and a high dose of baicalein (H-Bai) on iron accumulation and oxidative stress in mice induced by iron overload. (A to C) Assessment of iron deposition in the liver, spleen, and femur by Prussian blue staining. (D to G) Quantitative evaluation of the total iron content in the liver, spleen, tibia bone marrow, and bone shaft. (H) Detection of ROS levels in bone marrow using dihydroethidium (DHE) staining. (I) Quantitative analysis of DHE fluorescence intensity. (J) Detection of serum ferritin levels. (K) Measurement of serum MDA levels. (L) Evaluation of the ratio of GSH to GSSG in serum. *P < 0.05 vs. Ctrl; ^#^*P* < 0.05 vs. Fe.

Prussian blue staining showed that baicalein significantly alleviated iron overload-induced iron deposition in the liver, spleen, and bone marrow (Fig. 6A to C). Consistently, atomic absorption spectroscopy detection also revealed that iron dextran injection significantly increased iron content in the liver, spleen, bone marrow, and tibia diaphysis, while baicalein treatment decreased the iron content in these tissues (Fig. 6D to H). Staining with the fluorescent probe dihydroethidium found that excess iron caused a significant increase in bone marrow ROS, which was reduced by high-level-baicalein treatment (Fig. 6G and I). Serum biochemical results revealed that a high dose of baicalein markedly reversed the iron overload-induced increases in serum ferritin and MDA levels, as well as the decrease in the GSH/GSSG ratio (Fig. 6J to L).

### Baicalein activated the Nrf2/GPX4 signaling in iron-overloaded mice

**Fig. 7.**
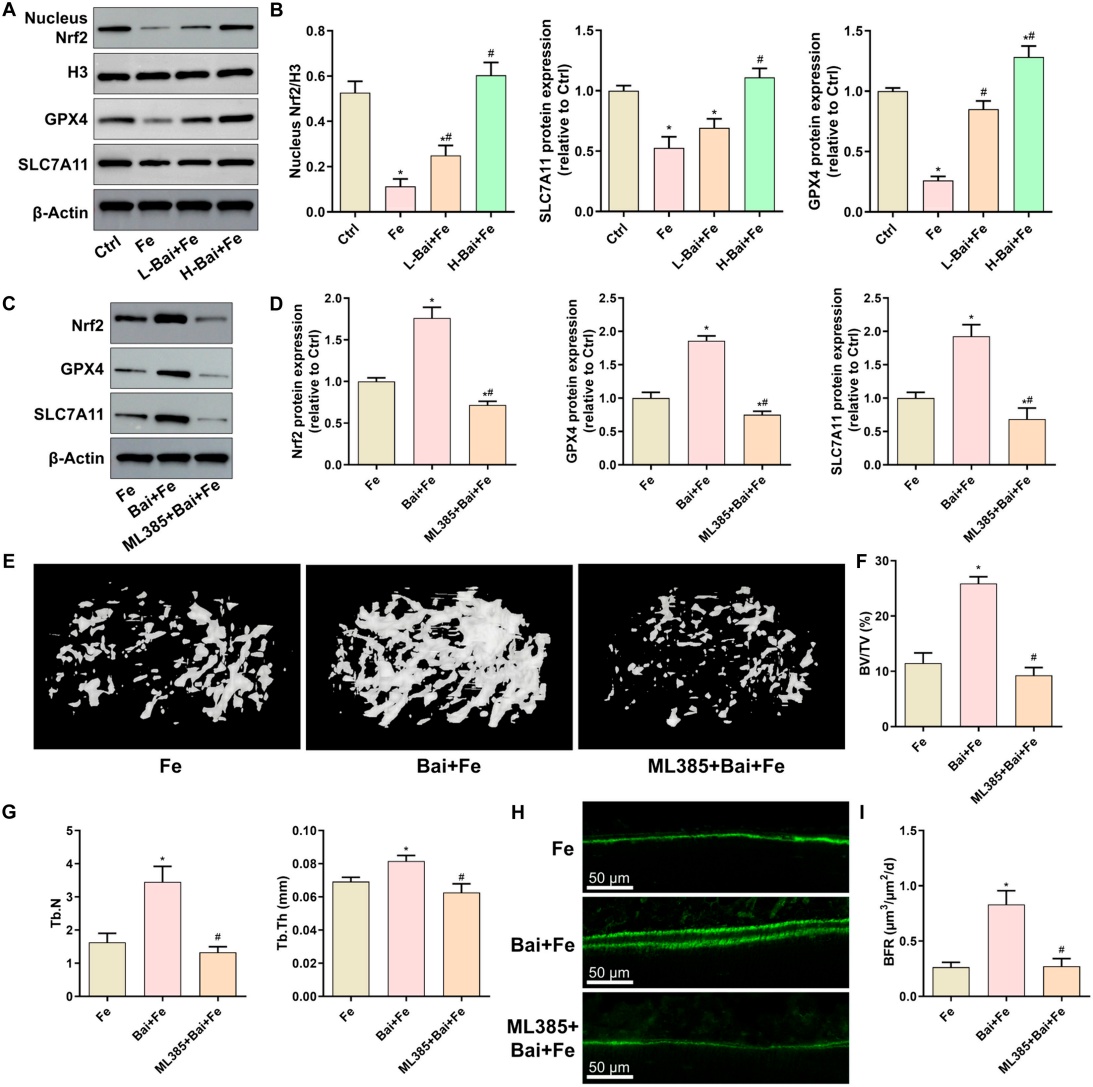
Evaluation of the effects of baicalein on the Nrf2/GPX4 pathway in the bone tissue of mice induced by iron overload. (A) Protein expression of nuclear Nrf2, GPX4, and SLC7A11 in the femur detected by Western blot. (B) Quantitative analysis of the gray values of nuclear Nrf2, GPX4, and SLC7A11 protein bands. (C) Protein expression of total Nrf2, GPX4, and SLC7A11 in the femur detected by Western blot. (D) Quantitative analysis of the gray values of total Nrf2, GPX4, and SLC7A11 protein bands. (E) Three-dimensional reconstruction images of the femoral trabecular bone. (F and G) Quantitative analysis of the femoral microarchitecture. (H) Evaluation of bone formation using calcein double labeling. (I) Quantitative analysis of BFR. *P < 0.05 vs. Ctrl in (A) and (B); ^#^*P* < 0.05 vs. Fe in (A) and (B); *P < 0.05 vs. Fe in (C) to (I); ^#^*P* < 0.05 vs. Bai+Fe in (C) to (I).

Evaluation of proteins related to the Nrf2/GPX4 signaling pathway in femoral tissue showed that baicalein significantly inhibited the iron dextran-induced down-regulation of nuclear Nrf2, GPX4, and SLC7A11 proteins (Fig. 7A and B). To further demonstrate whether baicalein prevents bone loss by activating the Nrf2/GPX4 signaling in iron-overloaded mice, we administered the Nrf2 inhibitor ML385 to these mice. The results showed that ML385 completely abolished the promoting effects of baicalein on the protein expression of Nrf2, GPX4, and SLC7A11 in iron-overloaded mice (Fig. 7C and D). Micro-CT results showed that the inhibition of Nrf2 prevented baicalein from reversing the iron dextran-induced bone loss in mice (Fig. 7E to G). Bone histomorphometry also showed that ML385 injection eliminated the inhibitory effects of baicalein on the iron overload-induced decrease in bone formation (Fig. 7H and I). These data reveal that baicalein prevents iron overload-induced bone loss and reduced bone formation by activating the Nrf2/GPX4 signaling pathway.

## Discussion

In this study, we demonstrated that baicalein mitigated iron overload-induced ferroptosis and restored osteogenic capacity in osteoblasts through activation of the Nrf2/GPX4 axis and that oral baicalein prevented iron accumulation-driven trabecular deterioration and biomechanical weakening in vivo; genetic (siNrf2) and pharmacologic (ML385) inhibition of Nrf2 abolished these benefits, supporting pathway specificity. Together, these data position baicalein as a candidate antiferroptotic therapy for iron overload-related bone disorders.

Iron is a reactive metal that alternates between Fe^2+^ and Fe^3+^ states, donating or accepting electrons to catalyze the Fenton reaction, thereby generating ROS [31]. The present study demonstrated that excess iron significantly induced ROS production in osteoblasts, whereas supplementation with baicalein markedly reduced cellular ROS levels. As a flavonoid, baicalein belongs to a class of natural products well-known for their potent free radical scavenging capacity [32]. As early as 1993, Hamada et al. [33] conducted a detailed in vitro investigation using electron spin resonance spectroscopy, revealing that baicalein quenches 1,1-diphenyl-2-picrylhydrazyl, superoxide, and hydroxyl radicals in a dose-dependent manner. Later, Gao et al. [34] reported that baicalein reduced the signal intensity of hydroxyl radicals, superoxide anions, and *tert*-butyl peroxyl radicals in 5,5-dimethyl-1-pyrroline-*N*-oxide spin adducts in a concentration-dependent pattern. In addition to its free radical scavenging ability, baicalein also exhibits strong iron-chelating properties. It is reported that baicalein has a higher binding affinity for ferrous ions than that of ferrozine, a known ferrous iron chelator [15]. Consistent with these findings, the present study also observed that baicalein significantly reduced Fe^2+^ levels in iron-exposed osteoblasts. Although we cannot entirely rule out contributions from other mechanisms (e.g., modulation of iron uptake or iron efflux), the existing literature strongly supports iron chelation as a primary mechanism. Beyond in vitro evidence, our in vivo data further demonstrated that baicalein markedly alleviated iron accumulation and oxidative stress in iron-overloaded mice. Similarly, a previous study showed that feeding mice with diets supplemented with baicalin (the glucuronide form of baicalein) resulted in dose-dependent reductions in hepatic iron content and lipid peroxidation, along with increased fecal iron excretion, indicating that baicalin chelates iron in vivo and facilitates its elimination via feces [18,35,36]. These results collectively suggest that baicalein protects against oxidative damage through dual mechanisms: scavenging free radicals and chelating iron.

Ferroptosis is closely associated with the pathogenesis and progression of osteoporosis, particularly as ferroptosis in osteoblasts leads to an imbalance in bone remodeling [30,37]. Substantial evidence indicates that aging, estrogen deficiency, and various secondary factors, such as diabetes, glucocorticoid, and obesity, can trigger ferroptosis in osteoblasts, thereby contributing to the development of osteoporosis [38–41]. As a form of iron-dependent cell death, ferroptosis is frequently observed in conditions of iron overload [23,42]. It has been established that iron overload induces ferroptosis in osteoblasts [25], suggesting that this mechanism may underlie iron overload-related bone disorders such as osteoporosis. Given baicalein’s notable free radical scavenging and iron-chelating abilities, its capacity to inhibit ferroptosis is highly plausible. Indeed, Xie et al. [43] screened 143 natural compounds for their protective effects against erastin-induced cell death and found that baicalein demonstrated the most potent protective effect against ferroptosis. The core hallmarks of ferroptosis include depletion of intracellular GSH and accumulation of lipid peroxides. Our study revealed that baicalein significantly counteracted the decrease in the GSH/GSSG ratio and the down-regulation of GPX4 caused by iron overload and prevented iron-induced accumulation of lipid peroxides. These findings suggest that baicalein acts as a protective agent against ferroptosis in osteoblasts.

The transcription factor Nrf2 orchestrates cellular defenses against redox imbalance and lipid peroxidation, partly through regulating the expression of 2 key gatekeepers of ferroptosis: SLC7A11 and GPX4 [44]. As such, the Keap1/Nrf2 pathway is recognized as a master regulator of ferroptosis in osteoblasts [45]. Under stress conditions, Nrf2 dissociates from the Keap1–Nrf2 complex, translocates into the nucleus, and initiates the transcription of genes containing antioxidant response elements [46]. Previous studies have reported that Nrf2 activation protects osteoblasts from ferroptosis in bone tissue and alleviates osteoporosis [28,47]. Consistently, we observed that under iron overload conditions, baicalein restored nuclear Nrf2 levels and the expression of downstream targets GPX4 and SLC7A11. Both genetic and pharmacological inhibition of Nrf2 abolished the cytoprotective and pro-osteogenic effects of baicalein. These findings suggest that baicalein prevents ferroptosis in osteoblasts via activation of the Nrf2/GPX4 pathway.

Clinically, iron overload conditions, such as transfusion-induced iron overload in thalassemia and hereditary hemochromatosis, are strongly associated with low bone mass and increased fracture risk [48,49]. Current treatment options (e.g., iron chelators, phlebotomy, and anti-resorptive agents) fail to simultaneously address iron overload and bone damage. Baicalein has undergone human safety and pharmacokinetic studies, which indicate no significant side effects even at high doses [50,51]. Our study demonstrates that baicalein not only prevents bone loss by protecting osteoblasts from ferroptosis but also effectively reduces systemic iron storage. Although beyond the scope of this work, baicalein’s known anti-osteoclastogenic effects may synergistically contribute to its overall bone-protective actions in iron overload conditions. These findings suggest that baicalein is a promising therapeutic agent for iron overload-related bone disorders. Although clinical trials are warranted, the dose of baicalein used in our study was extrapolated from clinically tolerated doses in humans, thereby supporting the potential feasibility of its clinical application.

In summary, this study provides the first definitive evidence that baicalein effectively inhibits iron overload-induced ferroptosis in osteoblasts by activating the Nrf2/GPX4 signaling pathway, thereby promoting bone formation and preventing bone loss. Our findings not only elucidate the mechanism by which baicalein functions as a novel ferroptosis inhibitor in bone protection but also highlight its role as a “dual-function” therapeutic strategy—combining iron chelation and anti-bone-loss capacities. Given its favorable safety profile and existing human pharmacokinetic data, our results provide strong preclinical evidence supporting the clinical translation of baicalein for the treatment of iron overload-related bone diseases. Targeting the ferroptosis pathway, particularly via Nrf2/GPX4 activation by baicalein, represents a highly promising novel strategy for preventing and treating iron overload-induced bone loss.

## Methods and Materials

### Cell culture

The MC3T3-E1 osteoblastic cell line was purchased from the American Type Culture Collection. Osteoblasts were cultured in alpha minimal essential medium (Gibco, Grand Island, NY, USA) supplemented with 10% (v/v) fetal bovine serum (Gibco), 2 mM l-glutamine, and 1% penicillin–streptomycin (Beyotime, Shanghai, China) and incubated in a humidified atmosphere of 5% CO_2_ at 37 °C. To induce osteoblastic differentiation, ascorbic acid (50 μg/ml; Sigma-Aldrich, St. Louis, MO, USA) and β-glycerophosphate disodium salt hydrate (10 mM; Sigma-Aldrich) were added to the medium of MC3T3-E1 cells that had grown to confluence. The medium was replaced with fresh medium every 48 h. Based on previous publications, a cell model of iron overload was established using 50 μM iron dextran (no. I121239, Aladdin, Shanghai, China). Baicalein (no. B107324, Aladdin) was dissolved in dimethyl sulfoxide and diluted to the concentrations specified in the experimental protocol using the culture medium before use.

### Cell viability

After cell treatments, the culture supernatant was discarded and replaced with a medium containing the Cell Counting Kit-8 reagent, followed by incubation for 2 h at 37 °C. The absorbance at 490 nm was measured using a microplate reader (Thermo Fisher, MA, USA).

### ALP staining

After 7 d of induction of osteoblastic differentiation, cells were fixed with 4% paraformaldehyde for 20 min. The BCIP/NBT Alkaline Phosphatase Color Development Kit (no. C3206, Beyotime) was used to prepare the BCIP/NBT staining working solution according to the manufacturer’s instructions. After adding the working solution, cells were incubated at room temperature protected from light for 30 min and then washed twice with distilled water. Staining was observed and photographed using a stereomicroscope (JS26, NOVEL, Nanjing, China).

### ALP activity assay

After 7 d of induction of osteoblastic differentiation, cells were fully lysed with radioimmunoprecipitation assay (RIPA) lysis buffer (no. P0013B, Beyotime). The Alkaline Phosphatase Assay Kit (no. P0321S, Beyotime) was used according to the instructions; the detection buffer and a chromogenic substrate were added, followed by incubation at 37 °C for 10 min. After adding the reaction stop solution, the absorbance at 405 nm was immediately measured using a microplate reader. The ALP activity in the samples was calculated based on the absorbance values of the standards.

### Alizarin Red S staining

After 21 d of induction of osteoblastic differentiation, cells were fixed with 4% paraformaldehyde for 20 min. A 0.5% Alizarin Red S staining solution (pH 4.2; no. G1038, Servicebio, Wuhan, China) was added, and cells were stained at room temperature for 15 min before being rinsed thoroughly with tap water. Mineralized nodules were observed and photographed using a stereomicroscope, and the area of mineralized nodules was analyzed using the ImageJ software (National Institutes of Health, Bethesda, MD, USA).

### Fluorescent probe staining

For the detection of cellular Fe^2+^, ROS, mitochondrial superoxide, and lipid peroxidation, the fluorescent probes FeRhoNox-1 (no. GC20134, GlpBio, Montclair, CA, USA), DCFH-DA (no. G1706, Servicebio), MitoSOX Red (no. G1734, Servicebio), and C11 BODIPY (no. GC40165, GlpBio) were used for incubation, respectively. Cells were mounted with an antifluorescent quencher containing 4′,6-diamidino-2-phenylindole (DAPI; no. 0100-20, SouthernBiotech, Birmingham, AL, USA) and imaged under a fluorescence microscope (NE610, NOVEL). Fluorescence intensity was analyzed using the ImageJ software.

### Cellular Fe^2+^ content detection

After cell culture, cells were processed according to the instructions of the Ferrous Iron Content Assay Kit (no. BC5415, Solarbio, Beijing, China). The absorbance at 593 nm was measured using a microplate reader, and the Fe^2+^ content in the samples was calculated based on the absorbance values of the standards.

### Cellular MDA, GSH, and GSSG detection

After cell culture, the Lipid Peroxidation (MDA) Assay Kit (no. S0131S, Beyotime) was used to detect the MDA content in cells. The GSH and GSSG Assay Kit (no. S0053, Beyotime) was used to detect the levels of GSH and GSSG in cells, and the GSH/GSSG ratio was calculated.

### Transmission electron microscopy

After culturing cells with different interventions, cells were fixed with 2.5% glutaraldehyde for 10 min. Cells were harvested using a cell scraper and resuspended in 2.5% glutaraldehyde overnight at 4 °C. Cells were preprocessed according to standard procedures, including staining, dehydration, embedding, and ultrathin sectioning. The ultrastructure of mitochondria was observed using a transmission electron microscope (Hitachi, Tokyo, Japan).

### Cell transfection

Cells were transfected with siRNA targeting Nrf2 (siNrf2; GenePharma, Shanghai, China) to knock down Nrf2 gene expression. According to the manufacturer’s instructions, cells were treated with 100 pmol of siRNA for 6 h, and silencing efficiency was detected by Western blotting. The transfected cells were then subjected to group interventions.

### Animal model and grouping

Eight-week-old male C57BL/6 mice (Charles River, Beijing, China) were housed in a clean environment at approximately 24 ± 1 °C under a 12-h light/dark cycle. Mice had free access to food and water. As reported previously by us, an iron overload mouse model was established by intraperitoneal injection of iron dextran at a dose of 500 mg/kg body weight once per week [52]. Control mice were injected with an equal volume of saline. There were 8 mice per group. Mice were grouped and treated according to the following experimental protocols:

Experiment 1: Thirty-two mice were randomly divided into 4 groups (*n* = 8): the control group (Ctrl), iron overload group (Fe), low-dose-baicalein group (L-Bai+Fe), and high-dose-baicalein group (H-Bai+Fe). Iron-overloaded mice were treated with high or low doses of baicalein via gavage. The high dose of baicalein was calculated based on the maximum safe human dose from previously published clinical trials (approximately 8 mg/kg) using the following formula [50]: Animal dose (mg/kg) = human dose (mg/kg)/(animal *K*_m_/human *K*_m_), where the *K*_m_ for mice is 3 and that for humans is 37 [53]. The calculated high dose of baicalein was 100 mg/kg, and the low dose was defined as 1/10 of the high dose (10 mg/kg). Administration was performed daily via gavage.

Experiment 2: Twenty-four mice were randomly divided into 3 groups (*n* = 8): the iron overload group (Fe), baicalein group (Bai+Fe), and ML385 + baicalein group (ML385+Bai+Fe). The intervention for the baicalein group was the same as that for the high-dose-baicalein group in experiment 1. The mice in the ML385 + baicalein group received daily intraperitoneal injections of ML385 (30 mg/kg) concurrently with high-dose-baicalein treatment [54].

After 8 weeks of treatment for all groups, mice were anesthetized with isoflurane, and blood samples were collected. Following euthanasia, femurs, tibias, livers, and spleens were harvested. All protocols were approved by the Experimental Animal Ethics and Welfare Committee of the People’s Hospital of Longhua, Shenzhen.

### Micro-CT

Mouse femurs fixed in 4% paraformaldehyde for 24 h were scanned using a SkyScan-1176 CT scanner (Bruker, Kontich, Belgium). Acquired images were reconstructed 3-dimensionally using the matched software NRecon (Bruker). Morphometric analysis of the defined region of interest was performed using the CTAn software (Bruker) to obtain microstructural parameters, including BV/TV, Tb.N, Tb.Th, and Conn.Dn.

### Bone strength measurement

A materials testing machine (Dongri, Guangdong, China) was used to perform a 3-point bending test on tibias to evaluate biomechanical properties. Tibias were loaded at a rate of 5 mm per minute until fracture. The fracture cross-section of the tibia was approximated as an ellipse. The outer and inner diameters of the long and short axes of the fracture cross-section were measured using a stereomicroscope. Based on our previous publication [55], the structural properties of the tibia, including ultimate load and stiffness, were calculated by analyzing the load–displacement curve. The material strength of the tibia, including ultimate stress and elastic modulus, was calculated from the stress–strain curve.

### Histochemistry

Femurs were fixed and decalcified, embedded in paraffin, and sectioned into 5-μm-thick slices using a semiautomatic rotary microtome (Leica Biosystems RM2245, Nussloch, Germany). Osteoblasts were identified by hematoxylin and eosin (Beyotime) staining.

### Dynamic histomorphometry

Mice were injected intraperitoneally with calcein (35 mg/kg) 10 and 3 d before euthanasia. Femurs were then fixed, dehydrated, embedded in methyl methacrylate, and sectioned (50 μm). The distance between the 2 fluorescent labels was measured using the ImageJ software, and BFR/BS was calculated.

### Prussian blue staining

To observe iron deposition, paraffin sections of liver, spleen, and femur were stained with Prussian blue, followed by enhanced staining with 3,3′-diaminobenzidine and finally counterstaining of nuclei with hematoxylin. Stained samples were observed and photographed under a light microscope.

### ROS assay in bone tissue

As per the established method in our previous publication [55], mice were injected intraperitoneally with dihydroethidium (25 mg/kg; no. S0063, Beyotime) 24 h before euthanasia. After sacrifice, tibias were isolated and fixed in 4% paraformaldehyde solution at 4 °C for 24 h. Subsequently, decalcification was performed using a commercial rapid decalcification solution based on formic acid (no. G1107, Servicebio) for 48 h, followed by paraffin embedding of the tibias. Sections of 5-μm thickness were obtained, and nuclei were labeled with DAPI for 30 min. Images were captured using a fluorescence microscope. The ImageJ program was used to quantify and analyze the fluorescence intensity in the images.

### Tissue iron content detection

The ends of the tibia were cut off, and bone marrow was isolated by centrifugation. The bone marrow, tibia diaphysis, liver, and spleen were then placed in an oven at 180 °C for 6 h to dry, and the weight of the dried tissue was measured using an electronic balance. Dried samples were incinerated in a muffle furnace (TAISITE, Tianjin, China) at 600 °C for 6 h. Subsequently, concentrated nitric acid (65%) was added to the incinerated samples, which were incubated on a constant-temperature heating plate at 70 °C for 2 h for complete digestion. The digested solution was diluted with a 0.1% potassium chloride solution, and the iron content was detected by atomic absorption spectroscopy (Analytik Jena, Germany) and normalized to the dry tissue weight.

### Serum biochemical assays

Blood was collected via cardiac puncture under anesthesia. Serum was separated by centrifuging blood at 3,000 rpm for 10 min at 4 °C. The MDA Assay Kit (no. S0131S, Beyotime) was used to detect MDA levels in serum. The GSH and GSSG Assay Kit (no. S0053, Beyotime) was used to detect the levels of GSH and GSSG in serum, and the GSH/GSSG ratio was calculated. Serum ferritin levels were determined using the Mouse Ferritin ELISA Kit (no. E-EL-M0491, Elabscience, Wuhan, China).

### Western blotting

Total protein was extracted from cells or femoral tissue using RIPA lysis buffer. The Nuclear Protein Extraction Kit (no. G3742, Servicebio) was used to harvest nuclear protein. The protein concentration was determined using the BCA Protein Quantification Assay Kit (no. G2026, Servicebio). Proteins were separated by sodium dodecyl sulfate–polyacrylamide gel electrophoresis and transferred to polyvinylidene fluoride membranes, which were then incubated with 5% skim milk. Subsequently, membranes were incubated with specific primary antibodies, including RUNX2 (no. AF5186, Affinity), COL-I (no. AF7001, Affinity), ALP (no. DF6225, Affinity), 4-HNE (no. ab48506, Abcam), GPX4 (no. GB120010, Servicebio), SLC7A11 (no. GB115276, Servicebio), Nrf2 (no. GB113808, Servicebio), Keap1 (no. GB113747, Servicebio), histone H3 (no. GB11102, Servicebio), and β-actin (no. GB15003, Servicebio). The next day, membranes were washed 3 times with Tris-buffered saline containing 0.1% Tween 20 (TBST) and incubated with corresponding species-specific horseradish peroxidase-conjugated secondary antibodies (Servicebio) at room temperature for 1 h. After washing with TBST, protein bands were visualized and imaged using a chemiluminescence imaging system (SCG-W3000 PLUS, Servicebio). Protein bands were quantified using the Image J software.

### Statistical analysis

All quantitative data are presented as mean ± standard deviation. To compare differences between groups, one-way analysis of variance followed by Fisher’s least significant difference multiple comparison test was performed using the GraphPad Prism software (version 9.4.1). A *P* value of less than 0.05 was considered statistically significant.

## Ethical Approval

All animal experiments were approved and carried out following the Institutional Animal Care and Use Committee. No human subjects were involved in this experiment.

## Data Availability

Data will be provided upon request.
